# Assessing the Functional Status of Patients with Chronic Pain—Cross Cultural Adaptation and Psychometric Properties of the Serbian Version of the Pain Disability Questionnaire

**DOI:** 10.3390/ijerph18136911

**Published:** 2021-06-28

**Authors:** Aleksandar Knežević, Petar Čolović, Milica Jeremić-Knežević, Čila Demeši-Drljan, Dušica Simić-Panić, Randy Neblett

**Affiliations:** 1 Faculty of Medicine, University of Novi Sad, 21000 Novi Sad, Serbia; milica.jeremic-knezevic@mf.uns.ac.rs (M.J.-K.); cila.demesi-drljan@mf.uns.ac.rs (Č.D.-D.); dusica.simic-panic@mf.uns.ac.rs (D.S.-P.); 2 Medical Rehabilitation Clinic Clinical Centre of Vojvodina, 21000 Novi Sad, Serbia; 3 Department of Psychology, Faculty of Philosophy, University of Novi Sad, 21000 Novi Sad, Serbia; petar.colovic@uns.ac.rs; 4 Institute of Child and Youth Healthcare of Vojvodina, 21000 Novi Sad, Serbia; 5 PRIDE Research Foundation, Dallas, TX 75235, USA; randyneblett@pridedallas.com

**Keywords:** chronic pain, disability, disability evaluation, quality of life, psychosocial distress, function, physical functional performance, Pain Disability Questionnaire

## Abstract

The Pain Disability Questionnaire (PDQ) has established itself as a leading patient-reported outcome measure for assessing both mental and physical components of pain-related disability. The current study aimed to translate the PDQ into Serbian and validate its psychometric properties. Following a standard translation process, a total of 554 chronic pain patients (average age 55.37 ± 12.72 years; 375 (67.5%) females) completed the PDQ-Serb, Oswestry Disability Index (ODI), Short Form-36 (SF-36), pain intensity rating and a six-minute walk test (6MWT). Responsiveness was examined in a subsample of 141 patients who completed an inpatient rehabilitation program. The internal consistency of the PDQ-Serb was excellent (Cronbach α = 0.92) and test-retest reliability was favorable (ICC = 0.87). Factor analyses found a bifactor model to be the best fit (CFI = 0.97: TLI = 0.96: RMSEA = 0.05; SRMR = 0.03). Statistically significant Pearson’s coefficient correlations (*p* < 0.001) were found between the PDQ-Serb and ODI (r = 0.786), SF-36 Physical Components summary (r = −0.659), SF-36 Mental Components summary (r = −0.493), pain intensity rating (r = 0.572), and 6MWT (r = −0.571). Significant post-treatment improvements following inpatient rehabilitation were found with the PDQ-Serb (*p* < 0.001; effect size 0.431) and other clinical variables (*p* < 0.001; effect sizes from 0.367 to 0.536). The PDQ-Serb was shown to be a reliable and valid self-report instrument for the evaluation of pain-related disability.

## 1. Introduction

Chronic pain is a major health care problem in Europe, affecting one in five European adults [1]. It can significantly affect one’s ability to perform physical activities and often results in disability [2,3,4]. If significant disability issues are detected, additional treatment time should be provided in order to address those issues [5]. Evaluating and monitoring the influence of pain on patients’ functionality is, therefore, an important precondition for identifying disability issues, designing adequate treatment, and improving patients’ quality of life [6].

Since its introduction [7], the Pain Disability Questionnaire (PDQ) has established itself as one of the leading patient-reported outcome measures in the field of pain-related disability assessment. The original psychometric evaluation in the English version of the PDQ determined two lower-order factors (the Functional Status Component and Psychosocial Component), within one higher-order dimension representing the general construct of pain disability [7]. The Functional Status Component measures the effects of chronic pain on activities of daily living, and the Psychosocial Component measures psychological distress and decreased perceived quality of life as a result of one’s painful medical condition.

Psychometric validation studies in English, Korean, and Brazilian Portuguese have determined test-retest reliability coefficients of total PDQ scores between 0.94 and 0.98 and Cronbach’s alpha internal consistency between 0.86 and 0.96 [7,8,9]. Total score differences have distinguished between different subject groups with presumably different levels of disability (e.g., chronic pain, acute pain, and control subjects) [7,8]. The PDQ has shown strong associations with other patient-reported measures of perceived disability, physical/mental functioning, pain interference, quality of life, and pain severity [7,8,9,10,11,12]. Associations have also been found with objective markers of physical impairment in subjects with chronic pain conditions, including distance and speed in a 6 minute walk test [13] and length of disability following workers compensation injuries [14]. It has demonstrated responsiveness to rehabilitative treatment with significant reductions in total and subscale scores [7,14]. Total PDQ scores have also predicted one-year post-rehabilitation work return/retention and health-utilization outcomes [7] and one-year post-total knee arthroplasty pain, quality of life, and functional outcomes [10,12]. 

Though most previous studies have assessed total PDQ scores, some have also used the lower-order factors as subscale scores [7,12]. The specific predictive power of the two subscales has not been adequately addressed. Large correlations between scales and their assumed linear dependence on the higher-order factor (both leading to multicollinearity) further complicate the problem. A bifactor model, comprising uncorrelated general and specific factors [15], could be a way to treat this issue. However, such a model has not yet been tested on the PDQ.

The current study had five goals. The first was to translate the original English version of the PDQ into the Serbian language. The second was to validate the psychometric properties of the PDQ-Serb, including structural validity, internal consistency, test-retest reliability, in a sample of patients with chronic pain. Third, by examining the structural properties of the questionnaire, we attempted to explore the adequacy of the bifactor structure, which may allow use of both general and the specific dimensions simultaneously, while avoiding the threat of multicollinearity. Fourth, the convergent validity of the PDQ-Serb was evaluated by its association with other patient-reported outcome measures (PROMS), including the Oswestry Disability Index (ODI), Short Form-36 (SF-36), pain intensity rating, and an objective measure of function (e.g., six-minute walk test). Finally, the treatment responsiveness of the PDQ-Serb and the other clinical variables was evaluated in a subsample of patients who completed an inpatient rehabilitation program.

## 2. Materials and Methods

The present study was performed in two parts. First, the English version of the PDQ was translated into Serbian, then the Serbian version of the PDQ was psychometrically evaluated in a group of chronic pain subjects. This study was approved by the Ethical Board of Clinical Centre of Vojvodina (No 00-15/575). All subjects signed informed consent before they were enrolled in the study.

### 2.1. Translation Process

The process of translating the PDQ from English to Serbian was performed according to the recommendations of the International Society for Pharmacoeconomics and Outcomes Research (IPSOR) guidelines [16]. This procedure has been described in more detail by our group in previous studies [17,18]. First, three native Serbian speakers performed an English-to-Serbian translation, then reworked the questionnaire items until a consensus was reached. A native English speaker and a professional translator performed a Serbian-to-English back-translation. Discrepancies in the translations were reworded as necessary to insure that the original content was accurately translated to the Serbian language. Discrepancies were identified for items #1, #10 and #13. For items #1 and #13, the meaning of the Serbian translation was too broad, so additional descriptors were added in parenthesis in the Serbian version to better define “everyday activities and work inside and outside the home.” The backward translation of the VAS scale on Serbian item #10 was “I go to see the doctor once a week.” It was changed to “I go to see the doctor at least once a week.”

Following the initial translation process, a sample of 10 patients with chronic pain were recruited for a cognitive-debriefing task to evaluate their understanding and clarity of the PDQ items. These patients were recruited from the Medical Rehabilitation Clinic in the Clinical Centre of Vojvodina (Serbia). They all agreed to participate and signed an informed consent. This sample included both genders, a range of ages, and various education levels. All subjects reported good understanding of the items, so no additional changes to the translation were made. At this point, the translated version of the PDQ-Serb was finalized. It is available online at https://www.pridedallas.com/questionnaires/ (accessed on 21 June 2021).

### 2.2. Study Population

A total of 554 patients were recruited from a rehabilitation program at the Medical Rehabilitation Clinic of the Clinical Centre of Vojvodina (Serbia) to participate in this study. Data collection occurred between 2017 and 2019. All patients had been referred to the Medical Rehabilitation Clinic from primary, secondary and tertiary health care institutions in the province of Vojvodina. Each subject agreed to participate and signed an informed consent. 

Demographic characteristics of the subject sample and diagnoses/localization of the pain are shown in the Table 1. Patients with various chronic painful conditions were included: low back pain, cervical pain, knee pain, hip pain, ankle pain, shoulder pain, lateral epicondylitis, polyneuropathy, and complex regional pain syndrome (CRPS). The Budapest criteria was used for diagnosing CRPS [19]. Widespread pain was determined by the 1990 ACR criteria [20]. If patients presented with multiple pain sites but did not meet 1990 ACR criteria, they were classified into a “multiple pain locations” group. A fibromyalgia diagnosis was made if patients met the Wolfe et al. (2016) criteria [21]. Chronic pain was defined as pain lasting for 3 or more months. All diagnoses were established by at least two physicians (referring physician and the treating physician at the Medical Rehabilitation Clinic). Patients were excluded: if pain duration was less than 3 months; age <18; they demonstrated poor Serbian language comprehension; they had an ongoing application for full-time long-term disability insurance payments; or they were in current litigation about insurance issues linked to their chronic painful condition. Subjects who were already receiving disability insurance payments were deemed eligible for study participation.

Due to the intention to examine both the time-dependent and time-invariant psychometric features of the PDQ-Serb, the total 554-subject sample was split into two subsamples. Sample 1 comprised 480 participants aged 18 to 86 years (mean age 55.39 ± 12.95), of whom 329 (68.5%) were women. Structural validity and internal consistency were examined in this sample. Sample 2 included the remaining 74 participants aged 26 to 78 years (mean 55.19 ± 11.12), of whom 46 (62.2%) were women. This subgroup of 74 patients completed the PDQ a second time, 3–5 days later, for the purpose of a test-retest analysis. Patients during this 3–5 day period did not receive any new therapy whenever it was ethically possible. Samples 1 and 2 did not differ in gender or age (*p* > 0.05).

In addition, responsiveness was examined in a subset of 141 patients (out of the 480 participants in Sample 1) who completed an inpatient rehabilitation program. (the remaining 413 subjects from the 554-subject total sample participated in various levels of outpatient rehabilitation treatment). The inpatient subjects did not differ from the rest of the total sample in age (55.02 ± 12.71 vs. 56.36 ± 12.70 years, t = −1.073, *p* = 0.284), pain duration (60.21 ± 83.68 vs. 74.75 ± 109.09 months, t = −1.442, *p* = 0.151) or female gender 278 (67.3%) vs. 97 (68.8%), X^2^ = 0.106, *p* = 0.835).

An individualized rehabilitation treatment plan was designed for each inpatient. It consisted of progressive therapeutic exercises and a disability management program under the supervision of physical and occupational therapists, along with the application of therapeutic modalities and aquatic therapy. It should be noted that no psychotherapy was provided during the treatment program. The treatment lasted an average of 19.87 ± 5.70 days, 3 h per day, six days per week. 

### 2.3. Clinical Variables

A battery of patient-reported questionnaires was administered in paper form to each subject following the initial medical evaluation. The same battery was also administered following treatment completion to the sub-sample of patients who participated in the inpatient rehabilitation program. 

The Pain Disability Questionnaire (PDQ) is a brief, fifteen-item measure that produces a total functional disability score between 0 and 150. Each item is worded in the form of a question and rated by the respondent on a visual analogue scale, which is scored on a ten-point scale. Two subscales are available. The Functional Status Component (items 1, 2, 3, 4, 5, 6, 7, 12, and 13) has a maximum score of 90 and the Psychosocial Component (items 8, 9, 10, 11, 14, and 15) has a maximum score of 60. Three disability severity ranges have been proposed, including Mild/Moderate (scores of 0–70); Severe (scores 71–100); and Extreme (scores 101–150) [14].

The Oswestry Disability Index (ODI) has 10 items, scored from 0 to 100, which measure perceived disability from activities of daily living [22,23]. Higher scores indicate more severe disability. The Cronbach alpha reliability coefficient for this measure is 0.894.

The Short Form–36 (SF-36v2) was used to assess perceived quality of life. It consists of two summary measures: the Physical Components summary (SF-36v2–PCS; score range of 0 to 100) and the Mental Components summary (SF-36v2–MCS; score range of 0 to 100). Higher scores indicate better perceived physical and mental performance and capacities [24]. The Cronbach alpha reliability coefficients were 0.85 for PCS-SF-36 and 0.78 for MCS-SF-36. 

Each subject estimated current pain intensity on a numeric rating scale (NRS) from 0 (no pain) to 10 (the worst possible pain). 

A six-minute walk test (6MWT) was also completed before and after the rehabilitation program. The 6MWT is a physical performance evaluation tool related to activities of daily living [25,26]. Subjects walked at a self-selected speed, back and forth down a 30m long hallway for six minutes, after which the covered distance was recorded. Participants were allowed to use their walking aids during this test.

### 2.4. Statistical Procedure

The initial step of the data analysis comprised screening the data for deviations from normality (using the “psych” package in R statistical environment [27,28]) and for outliers (by the combined method for outlier detection as implemented in the “Outlier Detection” R package [28,29]). The measures of skewness were below the absolute value of 1 for all PDQ items, while none of the kurtosis coefficients exceeded the absolute value of 1.5, suggesting no major deviations from univariate normality [30]. Multivariate kurtosis measured by the normalized Mardia’s coefficient pointed to deviation from multivariate normality (kurtosis = 23.36); hence, we used the ML estimation method with 2000 bootstrap samples in SEM to handle non-normality in continuous data [31]. Since we detected a single outlier, whose exclusion did not substantially decrease multivariate kurtosis, we decided to keep the outlying case in the analysis.

Considering the PDQ structure models found in previous studies [7,10], four models were tested and compared using confirmatory factor analysis: (1) A one-factor model, including a single latent dimension loading on all items; (2) A two-factor model, comprising two latent dimensions—Functional and Psychosocial Components; (3) A hierarchical model, containing the Functional and Psychosocial Components as the two lower-order factors, saturated by a higher-order factor; (4) A bifactor model, with the Functional and Psychosocial Components as specific (group) factors and one general factor (loading on all PDQ items according to the presumptions of the bifactor model). The defining feature of the bifactor model is the independence (orthogonality) of the general and specific factors, whereby the general factor loads on all items and specific factors load on particular groups of items. The confirmatory factor analysis was performed with the “lavaan” statistical package in the R environment [28,32]. Maximum likelihood was used as the estimation method. Due to slight deviations from normal distribution, the parameters’ standard errors were estimated using 2000 bootstrap samples. The following model fit indices were used: CFI—comparative fit index, acceptable values over 0.95; TLI—Tucker–Lewis index, acceptable values over 0.95; RMSEA—root mean square error of approximation, acceptable values lower than 0.10; SRMR—standardized root squared mean residual—acceptable values below 0.08 or 0.10 [33,34]. Factor reliability was estimated by the omega coefficient [35]. 

Test-retest reliability was examined by calculating the intraclass correlations (ICC) [36]. The analyses were conducted for both the overall PDQ-Serb score and the two subscale scores. Guidelines recommended by Koo and Li (2016) were used for interpretation, where values less than 0.5 indicate poor reliability, between 0.5 and 0.75 indicate moderate reliability, between 0.75 and 0.9 indicate good reliability, and greater than 0.90 indicate excellent reliability [36].

Pearson’s coefficient was used to test for convergent validity, calculating the correlation coefficients (corrected for multiple comparisons) among the PDQ, the other questionnaires, and the 6MWT. Specifically, the ODI score was chosen as a validation “counterpart” for the PDQ overall score, the two SF-36 scales were intended to serve as validation measures for the two PDQ subscales, while the 6MWT was an objective validation measure. Cohen’s criteria were used to interpret the Pearson’s r coefficients: small (0.1–r–0.29); medium (0.3–r–0.49); and large (0.5–r–1) [37]. The statistical packages psych (for R) and JASP were used to calculate Pearson’s coefficients and test their differences [27,38]. 

Treatment responsiveness was evaluated in the following manner. Values of the PDQ, ODI, 6MWT, and SF-36 were recorded before and after inpatient rehabilitation treatment and compared via paired-samples t test. Effect sizes were estimated via Cohen’s d values, which were calculated according to the formula:

$$d=\frac{\left|M1-M2\right|}{\sqrt{\mathrm{SD}1^{2}+\mathrm{SD}2^{2}-2\mathrm{rSD}1\mathrm{SD}2}}$$

Cohen’s criteria were used for interpretation: 0.2 small effect, 0.5 moderate effect, 0.8 large effect [37].

## 3. Results

### 3.1. Descriptive Statistics

As shown in Table 2, the descriptive statistics revealed that responses to all PDQ items spanned from 0 to 10, with median values ranging from three (item 2) to seven (items 1, 4, 5, 6, 7, 9, 10, and 12). Skewness coefficients suggested no substantial asymmetry, either for the individual items or for the scale scores. Items 8, 11, 13, 14, 15, and to an extent item 1, had negative kurtoses slightly out of the acceptable range, with item 8 having the “flattest” distribution. Additionally, Shapiro–Wilk normality tests were calculated for both the individual items and the scale scores. The results pointed to deviations from normality in all variables.

### 3.2. Factor Analysis

As shown in Table 3, all models except Model 3 converged successfully in 35–48 iterations. While Model 3 converged, its parameters included negative variance estimates. This result may have been an outcome of multicollinearity caused by the sizeable correlations among the lower-order scales. Similarly, Model 2 showed large correlations among the lower-order scales.

The bifactor model had the best fit indices. Model comparisons revealed significant differences among models, with the bifactor model being superior to the other models. Its general factor was well-saturated, with factor loadings ranging from 0.44 to 0.79. The first specific factor loaded saliently (approximately >0.30) on three items (PDQ3, PDQ4, and PDQ6, with respective loadings 0.38, 0.52, and 0.30), and the second factor on the items PDQ14 and PDQ15 (loadings 0.61 and 0.64, respectively). Factor reliability was estimated by the omega coefficient as 0.88 for the general factor, 0.04 for the first specific factor, and 0.19 for the second.

### 3.3. Internal Consistency

Internal consistency was excellent for the PDQ total score (Cronbach α = 0.92) and PDQ-Functional Component scale (Cronbach α = 0.90), and acceptable for the PDQ- Psychosocial Component scale (Cronbach α = 0.82).

### 3.4. Test-Retest Reliability

As shown in Table 4, the ICC coefficients for the PDQ overall score, Functional Status Component score, and Psychosocial Component score were 0.87, 0.82, and 0.85, respectively, pointing to favorable test-retest reliability.

### 3.5. Convergent Validity

As shown in Table 5, convergent validity of the PDQ-Serb was examined by calculating the Pearson correlations among the PDQ-Serb scores and scores on the ODI, SF 36, and 6MWT. The correlations among all measures were statistically significant at *p* < 0.001. The PDQ-Serb total score showed the highest correlation with the ODI total score and the lowest correlation with the SF-36 Mental Component summary subscale. The two PDQ-Serb subscales showed high to moderate correlations with the other questionnaires. The ODI total score correlated more strongly with the 6MWT than the PDQ-Serb total or subscale scores (z = 3.93, *p* < 0.001.)

In addition to the correlations among the PDQ scales’ sum scores and the measures mentioned above, correlations were calculated for the bifactor model factor scores, a.k.a. the PDQ structural model comprising the bifactor general and specific factors. The two specific factors did not yield a correlation above 0.45 with any of the other relevant variables, and only three correlations were above 0.30 (between the specific factor 2 and SF-36 Mental Component scale, r = −0.40, *p* < 0.001; the specific factor 2 and PDQ Psychosocial Component scale, r = 0.44, *p* < 0.001; the specific factor 1 and PDQ Functional Component scale, r = 0.34, *p* < 0.001). Stronger (moderate) correlations were found between the general factor and SF-36 Physical Components scale (r = −0.63, *p* < 0.001), ODI total score (r = 0.73, *p* < 0.001), 6MWT test (r = −0.55, *p* < 0.001), and Pain intensity (r = −0.51, *p* < 0.001), respectively. The general factor’s correlation with the SF-36 Mental Component was moderate (r = −0.41, *p* < 0.001) and extremely low with pain duration (r = 0.090, *p* = 0.05.)

### 3.6. Responsiveness

In order to investigate the responsiveness of the PDQ, ODI, 6MWT and SF-36, 141 patients were tested at the beginning and at the end of the inpatient rehabilitation program. As shown in Table 6, this subgroup of patients had significantly higher scores on the PDQ, ODI, and lower scores on the 6MWT compared to the rest of the sample (*p* < 0.001) indicating more severe disability. All measurements showed significant improvement at the end of treatment, except for the PCS-SF-36, where improvement did not reach a significant level.

## 4. Discussion

Many people with chronic pain are restricted in performing activities of daily living [1,39]. Standardized and validated patient reported outcome measures (PROMs) can provide a convenient method for assessing the impact of chronic pain on patients’ activities and functionality [40]. The PDQ was designed to evaluate both psychosocial and functional components of disability due to chronic pain [7]. In the present study, the PDQ was successfully translated into Serbian and psychometrically validated.

Test-retest reliability and internal consistency of the PDQ-Serb were both found to be within acceptable ranges. Four structural models were tested with confirmatory factor analyses. A bifactor model was found to be the best fit. However, despite its favorable overall features, the structural and measurement properties of the two specific factors were somewhat questionable, based on poor reliability coefficients of the two dimensions. This result reaffirms the stability of the general factor of “pain-related disability,” while at the same time posing a question about the two lower-tier dimensions’ conceptual tenability, as well as their predictive and incremental validity.

In order to investigate convergent validity, PDQ-Serb scores were correlated with other patient-reported disability-related measures, including pain intensity, pain duration, SF-36 (a measure of physical and mental well-being), and ODI (a similar measure of perceived disability). As has been found in previous PDQ studies, correlations among the PDQ-Serb total score, Functional and Psychosocial Status Components scores, and ODI were significant and substantial (moderate) [7,14]. Correlations of similar size were found with the Functional Component summary of the SF-36, which confirms the results of previous studies that have investigated the relationship between PDQ scores and quality of life measures [8,9]. Interestingly, the correlation between the ODI and Functional Component summary of the SF-36 was larger compared to the PDQ-Serb. This was not expected, as the ODI was originally designed for patients with low back pain, and in our sample, less than 50% of the patients reported low back pain.

The correlation between the Mental Component summary of the SF-36 and both the PDQ-Serb and ODI were in a medium range and almost identical. This was not expected, because the PDQ was designed to evaluate both psychosocial and physical components of disability, while the ODI was only designed to evaluate physical disability [7]. In fact, the evaluation of both physical and psychosocial components of disability has been proposed as an advantage of the PDQ compared to other disability PROMs, including the ODI, as certain chronic painful conditions, such as fibromyalgia, demonstrate more severe impact on mental components of quality of life compared to physical components [41]. However, there was almost no difference between the PDQ-Serb and ODI correlations with patient-reported perception of mental health quality of life in the present study.

Pain intensity is just one contributing factor affecting self-perceived disability and physical performance. Other factors (e.g., psychological), may also impact these clinical dimensions [42]. It is necessary to separately evaluate pain intensity and functionality, as improvements in pain intensity do not necessarily lead to improvements in function [43]. However, a significant correlation was identified between pain intensity and the PDQ-Serb, confirming results found in previous studies [7,9,12].

Correlations between pain duration and PDQ-Serb scores (and all the other patient-reported measures) were weak. This result was unexpected because higher PDQ scores have previously been found to be associated with longer length of pain-related disability following work injuries [14]. It is unknown if longer pain duration in the present study sample was also associated with prolonged disability. Pain severity could be a more important factor for disability and life quality than pain duration [44,45].

A 6MWT, which has shown promise as an assessment tool in patients with chronic pain, was used as an objective measure of physical performance [46]. Though it has been found to be superior to other physical performance measurements [47], some researchers have detected discrepancies between 6MWT scores and perceived levels of activity [48]. It has been noted that chronic pain patients are often not fully aware of their physical activity level [6], and pain intensity often correlates better with PROMs than with physical performance measurements [43]. In the present study, however, both the PDQ-Serb and ODI showed strong correlations with the 6MWT, indicating a substantial connection between perceived and objective pain-related functional disability. The significant association between PDQ-Serb total scores and 6MWT confirms results found in a previous study [13]. Interestingly, correlations between the ODI and 6MWT were larger compared to those with the PDQ-Serb.

We detected statistically significant improvement in pain intensity and functionality, assessed by the PDQ-Serb, ODI and 6MWT, after inpatient rehabilitation treatment, indicating significant responsiveness of these measurements. Effect sizes were evaluated to compare each of these measures, since direct comparison of mean differences between measures is not possible due to different scoring systems. Effect sizes were in a large range for the ODI and pain intensity and in a medium range for the PDQ-Serb total score, PDQ-Serb subscale scores, 6MWT and Mental Component summary of the SF-36. Improvement in the Physical Component summary of the SF-36 was not significant. The reason for this could be the short time frame for SF 36 assessment, as this time period was designed to be four weeks, and the treatment program was 3 weeks [24]. In contrast to the original English PDQ study, we did not find treatment responsiveness of the PDQ to be superior to the ODI [7]. Also, compared to the original English PDQ study, treatment responsiveness effect sizes in the present study were lower for the PDQ-Serb and ODI [7]. The reason for the lower effect sizes in the present study may be due to differences in the study populations and treatment programs. The patient population used for treatment responsiveness in the present study was assigned to an inpatient rehabilitation program and may have been more chronically disabled with pain than the study population in the original English PDQ study who attended outpatient treatment. The inpatient program in the present study was of relatively short treatment duration (<three weeks). The treatment duration in the original English PDQ study was longer (an average of 160 total hours over 2 or more months (personal communication with last author RN)) [7]. It should also be noted that psychotherapy was a treatment component in the English study but was not part of the inpatient program in the present study.

### Limitations

The current study was not free from limitations. As with other studies of this kind, the results were based on a chronic pain sample from a single hospital system in Serbia, so these findings may not be generalized to the rest of the Serbian population. We were somewhat limited in our ability to assess the convergent validity of the PDQ-Serb with other similar measures of perceived disability. We used the ODI in the present study because it is one of the few available disability questionnaires available in the Serbian language. However, though it has been used in previous studies that included non-spinal pain patients population [7,18,49,50,51], the ODI was designed for patients with low back pain [22,23]. Less than half of our study subjects reported low back pain.

## 5. Conclusions

It is recommended that health care providers use a variety of assessment methods when evaluating pain-related disability, including clinical assessment, observation of physical functioning, and patient self-report [47,52,53]. The PDQ has been shown to be a reliable and valid self-report instrument for this purpose [7,9,54]. This was the first cross-cultural adaptation of the PDQ into the Serbian language and the first evaluation of the psychometric properties of the PDQ-Serb. Our results confirmed good reliability and internal consistency, which is in the line with other versions of the PDQ [7,9,54]. In addition, our results confirmed convergent validity of the PDQ-Serb via its significant correlations with the ODI and 6MWT. These results indicate strong psychometric properties of the PDQ-Serb. Although the PDQ-Serb did not show psychometric superiority over the ODI, we consider its ability to assess psychosocial components of disability in chronic pain patients a benefit when compared with the ODI. Future studies should investigate the advantages of synthesized PDQ and physical performance measurements, such as 6MWT, in the evaluation of the functionality in patients with chronic pain.

## Figures and Tables

**Table 1:** Demographic characteristics of the patient sample (*n* = 554).

Variable	Value
Average age (years ± SD (min-max))	55.37 ± 12.72 (18–86)
Gender n (%)	
Female	375 (67.7)
Male	179 (32.3)
Education n (%)	
<9 yrs of education	119(21.5)
9–12 yrs of education	331(59.7)
>12 yrs of education	104(18.8)
Pain location/type n (%):	
Low Back Pain	
sciatica	219 (39.5)
localized	32 (5.8)
Cervical pain	
cervicobrachialgia	32 (5.8)
localized	7 (1.3)
Pain in two or more locations/pathologies	
Widespread pain	75 (13.5)
Multiple pain locations	69 (12.5)
Fibromyalgia	72 (13.0)
Localized extremity pain	
Shoulder pain	8 (1.4)
Epicondylitis	5 (0.9)
Hip pain	6 (1.1)
Knee pain	22 (4.0)
Ankle pain	4 (0.7)
Complex Regional Pain Syndrome	2 (0.4)
Polyneuropathy	1 (0.2)
Total	554 (100)

**Table 2:** Descriptive statistics (*N* = 480).

	Mean	SD	Median	Min	Max	SKEW	Kurtosis	SE	Shapiro-Wilk	*p*-Value of Shapiro-Wilk
PDQ1	6.16	2.51	7.0	0	10	−0.61	−0.03	0.11	0.94	<0.001
PDQ2	3.31	2.90	3.0	0	10	0.50	−1.03	0.13	0.90	<0.001
PDQ3	5.52	2.89	6.0	0	10	−0.32	−0.84	0.13	0.95	<0.001
PDQ4	6.08	2.45	7.0	0	10	−0.69	−0.09	0.11	0.93	<0.001
PDQ5	5.96	3.06	7.0	0	10	−0.51	−0.80	0.14	0.92	<0.001
PDQ6	6.69	2.50	7.0	0	10	−0.81	0.17	0.11	0.92	<0.001
PDQ7	6.30	2.55	7.0	0	10	−0.69	−0.10	0.12	0.93	<0.001
PDQ8	4.22	3.57	5.0	0	10	0.19	−1.43	0.16	0.88	<0.001
PDQ9	6.71	3.08	7.0	0	10	−0.70	−0.66	0.14	0.88	<0.001
PDQ10	6.26	2.55	7.0	0	10	−0.51	−0.37	0.12	0.95	<0.001
PDQ11	4.84	3.05	5.0	0	10	−0.23	−1.15	0.14	0.93	<0.001
PDQ12	6.60	2.82	7.0	0	10	−0.60	−0.55	0.13	0.92	<0.001
PDQ13	5.49	3.29	6.0	0	10	−0.30	−1.13	0.15	0.92	<0.001
PDQ14	5.00	3.03	5.0	0	10	−0.09	−1.13	0.14	0.94	<0.001
PDQ15	4.10	3.11	4.0	0	10	0.23	−1.24	0.14	0.92	<0.001
PDQ total score	83.20	29.89	88.5	6	150	−0.49	−0.46	1.36	0.97	<0.001
Functional Status Component	52.09	18.61	54.0	0	90	−0.49	−0.40	0.85	0.97	<0.001
Psychosocial Component	31.19	13.36	33.0	0	60	−0.30	−0.71	0.61	0.98	<0.001

**Table 3:** Confirmatory factor analysis (*N* = 480).

Fit Measure	1-Factor Model	2-Factor Model	Hierarchical Model	Bifactor Model
CFI ^1^	0.89	0.91	0.91	0.97
TLI ^2^	0.88	0.90	0.90	0.96
RMSEA ^3^	0.09 (0.08–0.10)	0.08 (0.075–0.09)	0.08 (0.076–0.09)	0.05 (0.04–0.06)
SRMR ^4^	0.05	0.05	0.05	0.03
X^2^(df) ^5^	458.45(90)	389.33 (89)	389.33 (88)	179.87 (75)
AIC ^6^	32,531.74	32,464.62	32,466.62	32,283.17
BIC ^7^	32,656.96	32,594.01	32,600.18	32,470.99

^1^ comparative fit index, ^2^ Tucker–Lewis index, ^3^ root mean square error of approximation (boundaries of the 90% confidence interval are shown in brackets),^4^ standardized root squared mean residual, ^5^ chi square (degrees of freedom shown in brackets), ^6^ Akaike’s information criterion, ^7^ Bayesian information criterion.

**Table 4:** Intraclass correlations (ICC) (*n* = 74).

	Type	ICC	F	df1	df2	*p*	Lower Bound	Upper Bound
PDQ overall score	ICC1	0.869	14.215	73	74	0	0.813	0.910
Functional Status Component	ICC1	0.824	10.346	73	74	0	0.751	0.877
Psychosocial Component	ICC1	0.853	12.623	73	74	0	0.791	0.90

**Table 5:** Correlations among clinical variables (*N* = 480).

Variable	PDQ Total Score ^1^	PDQ-FSC			PDQ-PSC		ODI		PCS (SF 36)		MCS (SF 36)		6MWT		Pain Intensity	
PDQ-FSC ^2^	0.954	***	—													
PDQ-PSC ^3^	0.908	***	0.741	***	—											
ODI ^4^	0.786	***	0.800	***	0.643	***	—									
PCS (SF 36) ^5^	−0.659	***	−0.685	***	−0.529	***	−0.733	***	—							
MCS (SF 36) ^6^	−0.493	***	−0.407	***	−0.539	***	−0.490	***	0.174	***	—					
6MWT ^7^	−0.571	***	−0.585	***	−0.470	***	−0.718	***	0.559	***	0.393	***	—			
Pain intensity	0.572	***	0.556	***	0.509	***	0.576	***	−0.484	***	−0.334	***	−0.438	***	—	
Pain duration	0.119	**	0.136	**	0.075		0.140	**	−0.105	*	−0.068		−0.107	*	0.113	*

^1^ Pain Disability Questionnaire, ^2^ Pain Disability Questionnaire—Functional Status Component, ^3^ Pain Disability Questionnaire—Psychosocial Component, ^4^ Oswestry Disability Index, ^5^ Physical Components summary of the Short Form–36, ^6^ Mental Components summary of the Short Form–36, ^7^ 6-min walk test, * *p* < 0.05; ** *p* < 0.01; *** *p* < 0.001.

**Table 6:** Treatment responsiveness (*N* = 141).

	Before Rehab		After Rehab		Change				
Measure	Mean	SD	Mean	SD	Mean	SD	t	*p*	Effect Size
PDQ ^1^ total score	96.71	22.85	87.09	25.22	9.62	22.32	5.121	<0.001	0.431
PDQ-FSC ^2^	59.66	15.08	53.72	16.19	5.94	14.92	4.724	<0.001	0.398
PDQ-PSC ^3^	36.95	10.71	33.45	11.07	3.50	9.86	4.191	<0.001	0.370
ODI ^4^	49.56	15.69	44.06	14.60	5.50	10.93	5.934	<0.001	0.503
6MWT ^5^ (m)	265.24	101.44	291.13	110.20	−25.89	63.93	−4.775	<0.001	0.405
PCS-SF-36 ^6^	32.08	5.63	32.82	6.10	−0.74	4.87	−1.787	0.076	-
MCS-SF-36 ^7^	39.78	11.64	43.19	11.30	−3.41	9.29	−4.332	<0.001	0.367
Pain intensity on NRS ^8^	6.68	2.26	5.44	2.23	1.24	2.31	6.368	<0.001	0.536

^1^ Pain Disability Questionnaire, ^2^ Pain Disability Questionnaire—Functional Status Component, ^3^ Pain Disability Questionnaire—Psychosocial Component, ^4^ Oswestry Disability Index,^5^ 6-min walk test, ^6^ Physical Components summary of the Short Form–36, ^7^ Mental components summary of the Short Form-36, ^8^ Numeric Rating Scale 0–10.

## Data Availability

The data that support the findings of this study are available from the corresponding author upon reasonable request.
